# Multiple breath washout of hyperpolarized ^129^Xe and ^3^He in human lungs with three‐dimensional balanced steady‐state free‐precession imaging

**DOI:** 10.1002/mrm.26319

**Published:** 2016-07-12

**Authors:** Felix C. Horn, Madhwesha Rao, Neil J. Stewart, Jim M. Wild

**Affiliations:** ^1^ POLARIS, Unit of Academic Radiology, Department of Infection, Immunity & Cardiovascular DiseaseUniversity of SheffieldSheffieldUnited Kingdom; ^2^ Insigneo Institute of In‐Silico MedicineUniversity of SheffieldSheffieldUnited Kingdom

**Keywords:** hyperpolarized gas, ^129^Xe, ^3^He, multiple breath washout, lung function

## Abstract

**Purpose:**

To compare quantitative fractional ventilation measurements from multiple breath washout imaging (MBW‐I) using hyperpolarized ^3^He with both spoiled gradient echo (SPGR) and balanced steady‐state free precession (bSSFP) three‐dimensional (3D) pulse sequences and to evaluate the feasibility of MBW‐I with hyperpolarized ^129^Xe.

**Methods:**

Seven healthy subjects were scanned using ^3^He MBW‐I with 3D SPGR and bSSFP sequences. Five also underwent MBW‐I with ^129^Xe. A dual‐tuned coil was used to acquire MBW‐I data from both nuclei in the same subject position, enabling direct comparison of regional information.

**Results:**

High‐quality MBW images were obtained with bSSFP sequences using a reduced dose (100 mL) of inhaled hyperpolarized ^3^He. 3D MBW‐I with ^129^Xe was also successfully demonstrated with a bSSFP sequence. Regional quantitative ventilation measures derived from ^3^He and ^129^Xe MBW‐I correlated well in all subjects (*P* < 0.001) with mean Pearson's correlation coefficients of *r* = 0.61 and *r* = 0.52 for ^3^He SPGR‐bSSFP and ^129^Xe‐^3^He (bSSFP) comparisons. The average intersubject mean difference (and standard deviation) in fractional ventilation in SPGR‐bSSFP and ^129^Xe‐^3^He comparisons was 15% (28%) and 9% (38%), respectively.

**Conclusions:**

Improved sensitivity in MBW‐I can be achieved with polarization‐efficient bSSFP sequences. Same scan‐session 3D MBW‐I with ^3^He and ^129^Xe has been demonstrated using a dual‐tuned coil. Magn Reson Med 77:2288–2295, 2017. © 2016 The Authors Magnetic Resonance in Medicine published by Wiley Periodicals, Inc. on behalf of International Society for Magnetic Resonance in Medicine. This is an open access article under the terms of the Creative Commons Attribution License, which permits use, distribution and reproduction in any medium, provided the original work is properly cited.

## INTRODUCTION

Pulmonary function testing based on dynamic monitoring of exhaled tracer gases is becoming increasingly used in a clinical setting to monitor lung ventilation heterogeneity 1. Multiple breath washout (MBW) monitors the decay in concentration of resident N_2_ or washed‐in SF_6_ tracer gas at the mouth while subjects breathe oxygen or air over a period of several minutes. MBW is a sensitive marker of functional changes in the small airways (<2 mm), and obstructive lung disease is typically reflected in prolonged washout of tracer gas from the lungs. MBW‐derived parameters of ventilation heterogeneity have been shown to be more sensitive to early disease than flow‐dependent measures from spirometry in cystic fibrosis (CF) patients 2, 3 was found to be a sensitive marker of obstructive lung disease 4, 5, 6. Multiple theoretical approaches have been developed in attempts to derive regional information about different compartments of the lung from whole‐lung MBW signals [e.g., 7], but these are not capable of providing definitive regional quantitative information about lung ventilation.

MRI of hyperpolarized ^3^He and ^129^Xe gases offers unique insight into pulmonary ventilation and physiology 8, 9, 10. Various approaches have been proposed to quantify lung ventilation from imaging as a single number summarizing the total ventilated volume in the thorax—for example, percent ventilated volume 11 or by clustering ventilation into functional compartments 12. However, the major disadvantage of these methods is the sacrifice of regional information. An alternative approach is to directly convert image intensity from single‐breath ventilation‐weighted images into regional volume fraction with knowledge of the inhaled gas volume 13. Nevertheless, the quantitative interpretation of lung ventilation by this method is affected by spatial variations in the transmit/receive B_1_ field of the radiofrequency (RF) coil, T_1_ decay, and weighting of the signal by RF depolarization.

Dynamic imaging of gas wash‐in over multiple breaths of hyperpolarized ^3^He MRI was demonstrated as a quantitative method for measuring regional ventilation in guinea pig lungs 14. This method was subsequently improved and shown to be feasible for use in larger species, including humans 15, 16. Additionally, in humans, continuous spectroscopic acquisition during MBW has been demonstrated using residual ^3^He gas in the lungs after ventilation imaging 17. This methodology was recently extended to an MBW‐I technique capable of producing quantitative ventilation maps covering the whole of the lungs 18.

Quantitative methods use either MR signal build up (gas wash‐in) or decay (wash‐out) to derive the fractional ventilation (fraction of gas volume turned over in each breath) from the amount of signal change in each voxel. These methods compensate for the effects of hyperpolarized gas T_1_ decay, RF depolarization, and RF coil sensitivity and thus can provide fully quantitative measures of regional lung ventilation.

Previously, MBW‐I has been implemented using fast low flip angle spoiled gradient echo (SPGR) sequences with a single dose of hyperpolarized ^3^He 18. However, the scarcity and current high cost of ^3^He necessitates the use of reduced gas doses or a different gas isotope. This work evaluates three‐dimensional (3D) MBW‐I with balanced steady‐state free precession (bSSFP) sequences in order to maximize the use of the finite available polarization. Here, quantitative ventilation information derived from bSSFP sequences (with a 50% reduction in ^3^He gas dose) is validated against previously presented SPGR methods. 3D MBW‐I with hyperpolarized ^129^Xe has been challenging because of its approximately three‐fold lower Larmor frequency when compared with ^3^He. Recent advances in polarizer technology 19, 20 and optimized bSSFP imaging 21 have facilitated high‐quality ventilation imaging with ^129^Xe 22. Here, these gains in polarization and signal efficiency from bSSFP sequences are also exploited to demonstrate 3D MBW‐I with hyperpolarized ^129^Xe.

## METHODS

### Subjects

Seven healthy subjects between the ages of 24 and 38 years and with an FEV_1_ in the range of 82%–126% predicted (Table 1) were scanned under written informed consent and agreement of the National Research Ethics Service (United Kingdom).

**Table 1:** Subject Demographics and Study Results.

Subject	Sex	Age, y	Weight, kg	FEV_1_, %pred^a^	Study 1 Sequence	SNR^d^	Fractional Ventilation, *r,* Mean ± SD	Tidal Volume, mL	Global Turnover^e^	Images Used to Fit Fractional Ventilation	Voxel‐by‐voxel Correlation Coefficient[Link]	Bland–Altman Analysis, MD (SD) in %	Study 2 Imaging Nucleus	SNR^d^	Fractional Ventilation, *r,* Mean ± SD	Tidal Volume, mL	Global Turnover	Images Used to Fit Fractional Ventilation	Voxel‐by‐voxel Correlation Coefficient	Bland–Altman Analysis[Link], MD(SD) in %
1	F	32	61	116	SPGR	186	0.28 ± 0.09	627	0.22	4	0.58	30.1 (38)	^129^Xe	116	0.30 ± 0.08	740	0.30	3	0.59	38 (34)
					bSSFP	446	0.24 ± 0.11	602	0.21	4			^3^He	191	0.20 ± 0.07	450	0.19	4		
2	M	24	77	108	SPGR	48	0.33 ± 0.10	915	0.25	3	0.62	21.7 (27)	^129^Xe	48	0.35 ± 0.09	1110	0.31	4	0.46	25 (26)
					bSSFP^b^	27	0.28 ± 0.11	1103	0.26	3			^3^He	163	0.26 ± 0.07	920	0.27	3		
3	M	30	75	82	SPGR	96	0.25 ± 0.09	919	0.18	5	0.68	2.6 (23)	^129^Xe	102	0.30 ± 0.08	1150	0.27	3	0.81	2 (18)
					bSSFP	233	0.21 ± 0.08	937	0.21	5			^3^He	114	0.26 ± 0.07	980	0.28	4		
4	M	28	84	108	SPGR	147	0.22 ± 0.07	826	0.21	3	0.73	3.9 (23)	^129^Xe	121	0.24 ± 0.08	830	0.22	4	0.45	69 (45)
					bSSFP	240	0.22 ± 0.08	937	0.19	4			^3^He	178	0.11 ± 0.05	620	0.18	4		
5	M	28	79	108	SPGR	53	0.19 ± 0.09	589	0.11	4	0.40	28.7 (42)	^129^Xe	39	0.19 ± 0.08	685	0.14	3	0.28	12 (69)
					bSSFP	196	0.12 ± 0.06	523	0.13	5			^3^He	145	0.18 ± 0.07	500	0.15	4		
6	F	38	80	126	SPGR	82	0.47 ± 0.08	1206	0.35	3	0.56	7.6 (19)	—	—	—	—	—	—	—	—
					bSSFP^c^	202	0.47 ± 0.07	1265	0.34	4										
7	M	28	75	98	SPGR	60	0.24 ± 0.08	1132	0.22	3	0.67	11.6 (21)	—	—	—	—	—	—	—	—
					bSSFP^c^	82	0.26 ± 0.07	1048	0.23	4										

a Values were calculated according to Quanjer et al. 34.

b For ^3^He bSSFP acquisition, twice the in‐plane resolution and a gas dose of 200 mL ^3^He were used.

c bSSFP imaging was performed using a flip angle of 7° with 200 mL ^3^He.

d SNR = signal to noise ratio (standard deviation of noise divided by mean signal from the lungs) of the first image acquired during MBW‐I.

e Global fractional ventilation derived from the ratio of mean tidal volume (measured by pneumotachograph) to mean inspiratory lung volume (calculated from images).

MD = mean difference (expressed as the coefficient of variation) and SD = standard deviation (expressed in percent).

Pearson's correlation coefficient (P < 0.001 in all cases).

This study was divided into two parts. In study 1, all seven subjects underwent ^3^He MBW‐I with 3D SPGR and bSSFP sequences. In study 2, five of the seven subjects also underwent ^3^He and ^129^Xe MBW‐I using 3D bSSFP sequences to compare results with the two inert gas isotopes.

### Hardware

All MRI examinations were performed on a GE Signa HDx 1.5T scanner (GE Healthcare, Milwaukee, Wisconsin, USA). ^3^He was polarized to approximately 25% using a commercial spin‐exchange optical pumping polarizer (GE Healthcare, Amersham, United Kingdom). ^129^Xe (comprising approximately 86% of the xenon mixture) was polarized to approximately 25% using a custom‐built spin‐exchange optical pumping polarizer 19. For study 1, a flexible transmit/receive vest coil (CMRS, Brookfield, Wisconsin, USA) tuned to the ^3^He Larmor frequency (48.62 MHz) was used. For study 2, a custom‐built flexible dual‐tuned transmit‐receive coil tuned to both ^3^He and ^129^Xe Larmor frequencies (48.62 and 17.65 MHz) 23 was used, allowing a direct comparison of quantitative ventilation maps from the two nuclei without the need for position changes between scans or image registration techniques. During MBW‐I, gas flow at the mouth was recorded using a RSS 100HR pneumotachograph (Hans Rudolph, Shawnee, Kansas, USA).

### Imaging

Parameters for the 3D MBW‐I sequences for studies 1 and 2 are summarized in Table 2. Hyperpolarized gas doses were topped up to 1 L with N_2_ and inhaled from functional residual capacity 18. Flip angles were considerably lower than the optimum value for a single‐breath‐hold 3D bSSFP static ventilation imaging sequence at the chosen resolution (flip angle ≈ 22°), because washout is monitored over multiple breaths and therefore some longitudinal magnetization must be preserved. For study 1, two thirds of the optimum flip angle was chosen (flip angle = 14°) for bSSFP imaging, and for study 2, a higher gas dose and one third of the optimum flip angle (flip angle = 7°) was used for both ^3^He and ^129^Xe (Table 2).

**Table 2:** MBW‐I Parameters for Study 1 and Study 2.

Parameter	Study 1		Study 2	
	SPGR (^3^He)	bSSFP (^3^He)	bSSFP (^3^He)	bSSFP (^129^Xe)
Pulse repetition time, ms	2.5	1.6	1.4	2.9
Echo time, ms	0.75	0.6	0.4	0.9
Field of view, cm	38 × 30.4	38 × 30.4	38 × 30.4	38 × 30.4
Acquisition matrix, mm	32 × 26 × 26	32 × 26 × 26	32 × 26 × 26	32 × 26 × 26
Voxel size, mm	12 × 12 × 10	12 × 12 × 10	12 × 12 × 10	12 × 12 × 10
Scan time, s	1.7	1.1^a^	0.9	2.0
Slice thickness, mm	10	10	10	10
Bandwidth, kHz	32.3	166	166	16.1
Flip angle	1°	14°	7°	7°
RF pulse envelope	Gaussian	Gaussian	Hard pulse	Hard pulse
Pulse width, μS	500	500	200	200
Dose of hyperpolarized gas, mL	200	100^a^	200	600

a In subject 2 ^3^He bSSFP imaging was performed using 200 mL of hyperpolarized ^3^He to demonstrate the feasibility of acquiring images with double the in‐plane resolution (64 × 51 × 26), resulting in a voxel size of 6 × 6 × 10 mm and an acquisition time of 2.1 s.

### MBW‐I Protocol

MBW‐I breathing maneuvers and postprocessing of images was performed as described in detail 18. Briefly, the main components of the MBW‐I protocol were as follows. First, subjects were trained in the required breathing procedures both outside and inside the scanner before hyperpolarized gas imaging. Subjects inhaled a single dose of hyperpolarized gas from functional residual capacity. Upon inhalation, two sets of volumetric images were acquired during breath‐hold and the signal decay between corresponding images was analyzed to calculate a correction factor for compensation of non–washout‐related signal decay, including T_1_ decay and RF depolarization. Following the second acquisition, subjects began relaxed tidal breathing, interrupted by short breath‐holds for image acquisitions after each breathing cycle. All images were acquired with a fixed delay time of 4 s to allow one breathing cycle between acquisitions. Major airways were excluded from all calculations because a fractional ventilation *r* = 1 can be expected and no signal is received from the airways after the first volume turnover. Pneumotachograph recordings were used to exclude data when a tidal volume change of more than ±15% occurred.

**Figure 1:**
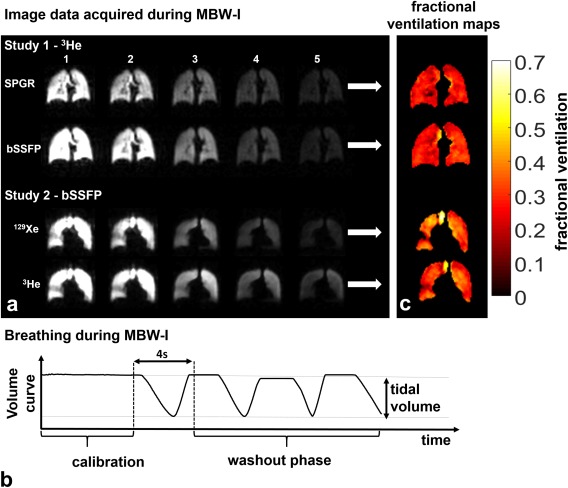
Representative results from MBW‐I. Comparison of ^3^He MBW‐I with SPGR with bSSFP sequences (subject 4) and comparison of ^129^Xe and ^3^He bSSFP MBW‐I (subject 3). Schematic lung volume curve during MBW‐I as derived from flow recordings at the mouth. Fractional ventilation maps of the slices shown in panel A. For all acquisitions, the time delay between images was fixed to 4 s to allow subjects to comfortably complete one breathing cycle.

Example washout data from a single slice for all subprotocols are shown in Figure 1. A typical time‐volume curve acquired with the flow meter during the protocol is shown in Figure 1. The resulting signal decay over all acquisitions was corrected for RF and T_1_ depolarization and fitted with an exponential least‐squares function to obtain gas turnover (fractional ventilation) on a voxel‐by‐voxel basis, as shown in Figure 1.

### Comparison

For both, subjects were not moved between imaging experiments, hence a voxel‐by‐voxel comparison of derived fractional ventilation maps could be performed without the need for image registration. We acknowledge that the lungs themselves do move, but the controlled breathing maneuver allows for a basic pixel‐wise comparison without image registration; to do so, fractional ventilation maps were masked with the intersection of the two maps. Pearson's *r* coefficient was calculated for the correlation between the two experiments, and Bland–Altman analysis was performed to determine the intrasubject mean difference and standard deviation as a percentage of the mean.

## RESULTS

The results of this study are summarized in Table 1.

### Study 1: ^3^He MBW‐I, SPGR Versus bSSFP

**Figure 2:**
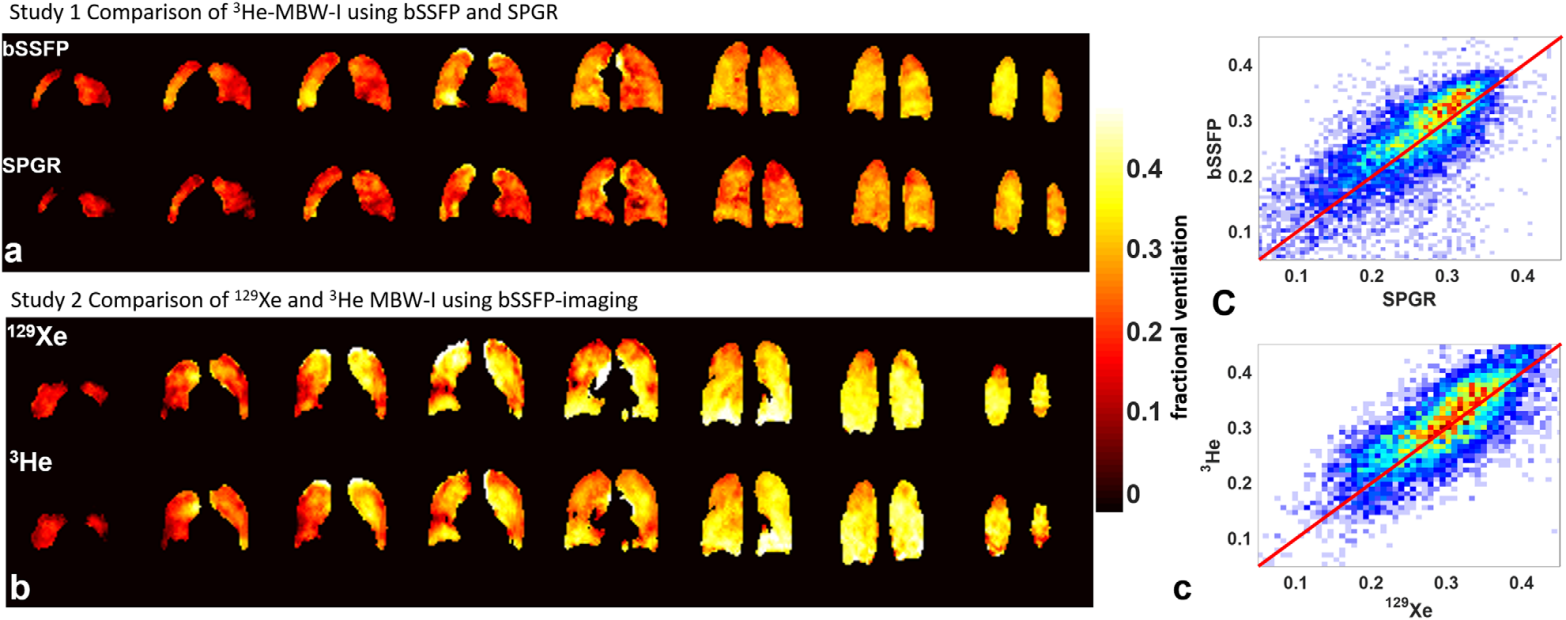
(**A**, **B**) Comparative fractional ventilation maps from ^3^He MBW‐I using bSSFP and SPGR sequences (subject 6) (A) and from ^129^Xe and ^3^He MBW‐I using bSSFP sequences (subject 3) (B). (**C**) Voxel‐by‐voxel correlation plots of the datasets in panel A (top, *r* = 0.56) and panel B (bottom, *r* = 0.81). The solid red line indicates the line of unity, and the color map represents the density of points, with blue <10 and red >30.

MBW‐I with ^3^He using both SPGR and bSSFP sequences was performed successfully in all seven subjects, and image quality was sufficient to generate fractional ventilation maps. ^3^He MBW images acquired from subject 4 with SPGR and bSSFP sequences are shown in Figure 1, alongside resulting fractional ventilation maps of the corresponding slices (Fig. 1). Similar values of fractional ventilation were found and good visual agreement of ventilation features was observed when comparing fractional ventilation maps derived from the two imaging sequences (Fig. 2). An average Pearson's correlation coefficient over all subjects of *r* = 0.61 (all *P* < 0.001) was determined. The mean intersubject difference (coefficient of variation) was found to be 15.2%, with a standard deviation of 27.6%.

**Figure 3:**
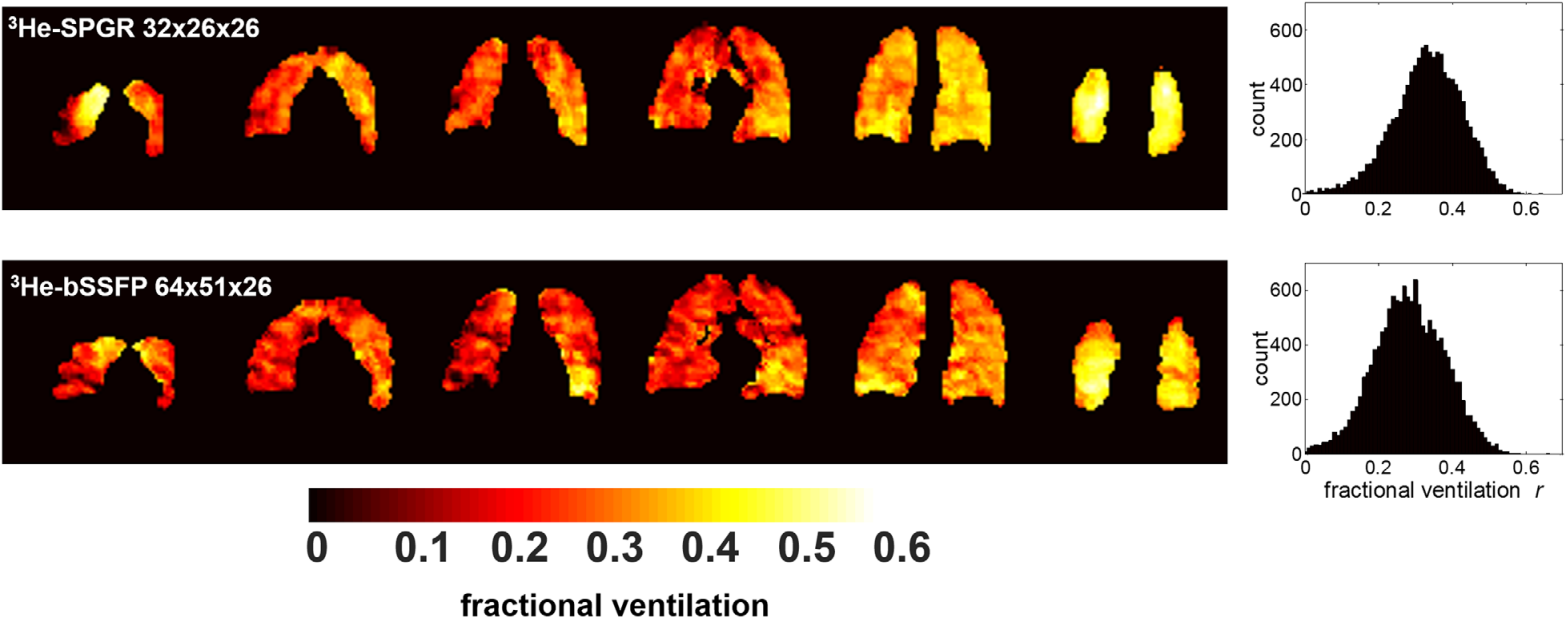
Comparison of fractional ventilation maps derived from subject 2 using low‐resolution SPGR and high‐resolution bSSFP imaging sequences with hyperpolarized ^3^He. The k‐space of the SPGR acquisition was zero‐filled before reconstruction to mimic the resolution of the bSSFP images. Fractional ventilation maps from selected slices (anterior to posterior) are shown, along with whole‐lung fractional ventilation histograms. Slight differences in the intensity of the maps from each acquisition result from using the same color map in both cases. Different average fractional ventilation values were obtained (SPGR average [standard deviation] *r* = 0.33 [0.10], bSSFP average [standard deviation] *r* = 0.28 [0.11]); see also the shift in peak position of the histograms.

To demonstrate the signal benefits associated with bSSFP sequences, an additional ^3^He MBW‐I dataset was acquired using a bSSFP sequence with a two‐fold increased spatial resolution (64 × 51 in‐plane, subject 2). The results of this scan are presented against a lower resolution SPGR scan for that subject in Figure 3. Fractional ventilation maps derived from images acquired at the two different resolutions showed similar features. Maps derived from the higher resolution scan appeared qualitatively slightly noisier than the lower resolution SPGR images; however, a significant Pearson's correlation of *r* = 0.62 (*P* < 0.001) was determined from a voxel‐by‐voxel comparison (after zero‐filling SPGR images to mimic the resolution of the bSSFP images).

### Study 2: MBW‐I with ^129^Xe

bSSFP MBW‐I with ^129^Xe was successfully performed in all investigated subjects and image quality was sufficient to derive quantitative fractional ventilation maps (mean values summarized in Table 1). Figure 1 shows raw images from ^129^Xe and ^3^He MBW‐I in subject 3, with corresponding fractional ventilation maps depicted in Figure 1. ^3^He and ^129^Xe fractional ventilation maps from slices covering the whole of the lungs are shown in Figure 2. Comparable values of fractional ventilation were obtained, with a trend of slightly increased *r* values for ^129^Xe, and good qualitative, visual agreement of features was observed when comparing ^3^He and ^129^Xe MBW‐I data, as illustrated in Figure 2 and the corresponding correlation plot in Figure 2. An average Pearson's correlation coefficient of *r* = 0.52 (with *P* < 0.001 in each individual case) was calculated. Bland–Altman analysis resulted in a mean intersubject difference (coefficient of variation) of 9.2% (biased towards larger values for ^129^Xe) with a corresponding intersubject standard deviation of 38.4%.

To validate MBW‐I, mean fractional ventilation was compared with the global lung volume turnover calculated from measurement of the tidal volume at the mouth and extraction of the lung volume from image segmentation. In both study 1 and study 2, a highly significant correlation of both independent measures was found with a mean Pearson's coefficient of *r* = 0.93 (*P* < 0.001) and *r* = 0.81 (*P* < 0.01), respectively.

## DISCUSSION

### Study 1: ^3^He MBW‐I, SPGR Versus bSSFP

The comparison of fractional ventilation values derived from ^3^He MBW‐I with SPGR and bSSFP sequences showed good agreement between sequences in all subjects. Considering the scarcity and expense of ^3^He, bSSFP MBW‐I presents a potentially more economically viable alternative to SPGR MBW‐I, requiring a 50% lower ^3^He gas dosage for images and fractional ventilation maps of comparable quality at the same spatial resolution. In addition, the 10% ^3^He: 90% N_2_ mix used for bSSFP scans better approximates air in the lungs in terms of density and diffusion coefficient 24. The feasibility of acquiring images with double the in‐plane resolution and the same gas dosage with the bSSFP sequence when compared with the SPGR sequence was also demonstrated in one subject. The lower resolution SPGR images in Figure 3 appear smoother than the higher resolution bSSFP images, which might be explained by the zero‐filling of SPGR data required to mimic equivalent resolutions (3). Nevertheless, the good voxel‐by‐voxel correlation between fractional ventilation data at both resolutions suggests that a voxel size of approximately 1 cm^3^ is sufficient to fulfill a major assumption of the washout model; that is, that ventilation is homogeneous within an image voxel. This assumption may hold in healthy subjects, but in patients with inhomogeneous ventilation (e.g., asthma), where ventilation heterogeneity begins in the small airways and can result in a patchy appearance on ^3^He ventilation images 25, it may be less applicable 26. Thus, an increase in spatial resolution of MBW‐I data should provide greater insight into the characteristic length scales of ventilation heterogeneity.

### Study 2: MBW‐I with ^129^Xe

3D MBW‐I with ^129^Xe has been demonstrated for the first time, made feasible by recent improvements in ^129^Xe polarization levels, and the efficient use of induced polarization afforded by bSSFP sequences. By comparison, in our previous preliminary work with SPGR sequences and lower ^129^Xe polarizations, image signal‐to‐noise ratio (SNR) was insufficient for multiple 3D acquisitions required for ^129^Xe MBW‐I 27. Here, comparable functional information was derived from MBW‐I datasets from both ^3^He and ^129^Xe nuclei, with similar ventilation heterogeneity observed. The voxel‐by‐voxel correlation of regional ^3^He and ^129^Xe fractional ventilation values resulted in a slightly lower average Pearson's correlation coefficient (*r* = 0.52, *P* < 0.001 in all cases) compared with the correlation between SPGR and bSSFP sequences for ^3^He. Only in subject 5 was the correlation coefficient noticeably lower than in other subjects. In addition, subject 5 was a former smoker, and some effects of lung obstruction were observed similar to subject 3 (Fig. 1A, study 2). It has been reported previously that xenon has a reduced ability to penetrate less ventilated airspaces when compared with helium 28, and therefore this might be expected to affect the measured fractional ventilation data. Additionally, xenon is a much denser (ρ_He‐N2_ = 1.143 kg/m^3^, ρ_Xe‐N2_ = 3.957 kg/m^3^) and less diffusive gas (D_He‐N2_ = 0.85 cm^2^/s, D_Xe‐N2_ = 0.10 cm^2^/s) 29, 30 than helium. In the case of ^129^Xe MBW‐I, the tracer gas itself comprises approximately 15% of the inspired lung volume, compared with approximately 2.5% for the ^3^He gas mix. The fact that ^129^Xe was mixed with nitrogen and not a lighter gas (such as ^4^He) to better approximate the physical properties of air is a limitation of this study. Nevertheless, the general absence of airway obstruction in the healthy subjects scanned in this study means this is expected to have a limited impact on the data presented here. Xenon signal loss by diffusive uptake into the blood and tissue was not considered when calculating fractional ventilation; it was assumed that the dissolved fraction (approximately 2%) was small enough to be negligible when compared with the fraction exhaled by ventilation 31.

The breathing pattern is the major limitation on accuracy and repeatability of quantitative fractional ventilation imaging. A trend of ^129^Xe MBW‐I resulting in higher mean fractional ventilation was observed, which could be linked to the consistently higher tidal volume measured with^129^Xe when compared with ^3^He experiments (Table 1). In the absence of ventilation defects, differences in mean fractional ventilation information from repeated experiments are likely to arise from an altered breathing pattern and/or different inspiratory levels. Those could potentially arise from differences in image acquisition time for ^129^Xe and ^3^He MBW‐I. The method also relies upon the subjects' ability to reproduce their breathing pattern between experiments. In order to reduce errors from variation in breathing within an experiment, images with a tidal and/or lung volume varying by more than ±15% of the mean were excluded. This rejection criterion resulted in a worst case propagated error in fractional ventilation of 21%. A passive volume delivery device could also be used to increase the reproducibility and accuracy of the subjects breathing pattern 32.

In addition, image SNR and the number of images used in fitting can influence the accuracy of the fractional ventilation parameter. In background work a Monte‐Carlo analysis of errors from MBW‐I was performed 33 with an initial SNR of approximately 60 using 3‐5 points for fitting. Assuming an average lung turnover of 0.15–0.35, the results showed an error of between 4% and 10% in the fractional ventilation parameter. An even lower error can be expected with higher image SNR values [see Horn 33 for reference]. Experimental parameters that affect the accuracy of the fit to fractional ventilation—including the number of images included in the fit and the respective image SNR—are provided in Table 1 for the different experiments performed.

In conclusion, bSSFP sequences allow efficient use of polarization and reduced gas dosage requirements when compared with SPGR for MBW‐I applications in humans. The achieved gains in SNR can alternatively be used to improve spatial resolution in MBW‐I. The feasibility of 3D MBW‐I with ^129^Xe has also been demonstrated successfully for the first time and was validated against ^3^He MBW‐I. In future work, it may be possible to identify sensitivity differences in MBW‐I with the different gases to characterize different physiological processes of obstructive lung diseases.
